# Evaluating the impact of a resident research program in general surgery

**Published:** 2017-06-30

**Authors:** Laura Allen, Kelly Vogt, Tina Mele, Michael Ott, Ken Leslie, Patrick Colquhoun

**Affiliations:** 1 London Health Sciences Centre, Division of General Surgery, Ontario, Canada

## Abstract

**Background:**

Programs of resident research have been found to improve research productivity. However, evidence of the success of these programs is lacking in a Canadian context. The objective of this study was to evaluate the impact of the introduction of a formal program of resident research at a single Canadian academic centre.

**Methods:**

Resident research activities were tracked over a 10-year period (Resident Research Day (RRD) presentations, abstract presentations, published articles). Activities were divided into pre (2002–2007) and post (2007–2012) resident research program implementation time frames. Differences in research productivity were compared between time frames. Surveys of resident attitudes towards research were administered prior to the program’s implementation in 2007, and following introduction of the resident research program in 2009 and 2015.

**Results:**

Overall, research productivity (abstracts, publications, and RRD presentations) increased between pre and post resident research program time periods, with a statistically significant increase in mean number of published abstracts. Resident attitudes towards research changed somewhat over time, with fewer residents supporting mandatory research in recent years.

**Conclusion:**

Implementation of a resident program of research resulted in a significant increase in research productivity. The setting of clear, modifiable, and achievable goals, as well as providing tools for research success, have contributed to the success of this program.

## Introduction

The medical field is constantly advancing with research conducted in every aspect of health care and the health care system.^1^–^3^ As such, it is of great importance that individuals in this field have a deep understanding of this ongoing research and are able to critically appraise the resulting literature.^1^ This knowledge and appreciation of research principles should allow for more appropriate translation of new information into clinical practice.^1^ Practical application of research methods through participation in research activities is one constructive way to develop this understanding and appreciation of the research field.^1^–^3^ For these reasons, many residency programs have developed a research curriculum at their institutions.^2^,^4^–^7^

In recent years, the support for formal resident research programs has grown. The Royal College of Physicians and Surgeons of Canada has an expectation that residents must be able to create, disseminate, and apply research as one of several key competencies.^8^ Similarly, in the United States (US), expectations for research requirements for all residents are outlined by The Accreditation Council for Graduate Medical Education.^9^ While a number of evaluations of resident research programs have been conducted across a variety of specialties, the vast majority of these studies have been conducted in the United States.^1^–^7^,^10^–^15^ In most cases, resident programs of research have demonstrated positive outcomes in terms of increased abstract submission, presentation at local and national conferences, 2,5–7,10,11,14–16 an increase in publication rates,^5^–^7^,^10^,^11^,^14^–^16^ and an improvement in the quality of publishing journals.^2^,^5^ In general, resident research programs have almost always improved productivity, sometimes quite dramatically. Similar evidence of the success of resident research programs is largely lacking in the Canadian context. The Canadian training paradigm differs considerably from the US with substantially fewer residency programs and training spots, as well as differing training requirements. Fewer restrictions on work hour in the Canadian residency environment^17^ may contribute to a greater amount of time spent in clinical activities, resulting in less time dedicated to education and research. Furthermore, research funding opportunities vary between the two countries^18^,^19^ potentially affecting the type and number of research opportunities available for Canadian residents. As such, data from US studies cannot be meaningfully applied to the Canadian population warranting specifically Canadian research.

The primary objective of this evaluation was to determine if the introduction of a program to support resident research in the division of General Surgery at a single Canadian academic centre has resulted in an increase in productivity as measured by conference presentations (published abstracts), peer-reviewed publications, and participation in the annual resident research day. A secondary objective was to explore changes over time in resident attitudes towards research.

## Methods

In 2007, the General Surgery Division at London Health Science Centre implemented its first formal resident research program to encourage and better support trainee research. This program was developed based on both a survey of residents about their attitudes towards their current research environment and on the annual resident feedback report prepared for the Division of General Surgery. Prior to the implementation of this program, research expectations for residents were unclear. Although there was a general expectation of scholarly activity and presentation at the annual resident research day, no specific requirements or objectives were delineated. As such, a formal program was developed in an effort to provide more structure, as well as to promote success in resident research.

At its inception, the goals of this new program were specific. We wanted to achieve: 1) 80% participation in Resident Research Day; 2) an improved quality of research; 3) an abstract submission rate of 50% to the Canadian Association of General Surgeons Canadian Surgery Forum (with a minimum expectation of a 30% acceptance rate); and 4) an increase in the number of papers submitted for publication. Resident research activities since the advent of this program have been tracked in order to evaluate these goals.

This program is guided by a resident research director who is a member of the Division of General Surgery. The resident research director meets with each resident twice a year to track progress, identify barriers to success, and provide assistance in overcoming these barriers. Residents also meet on an as needed basis with mentors to discuss progress on specific research projects.

In addition to mentor guidance, the research program also provides a number of strategies and materials to promote research success. As part of ongoing research education, academic half days are provided to residents and cover a rotating schedule of topics. A minimum of twice per year these sessions cover topics such as research methodology, critical appraisal of the literature, basic biostatistics, and technical aspects of health services research (i.e. library services, research ethics board, granting opportunities etc.). Attendance of these sessions is mandatory, as is attendance of a bi-monthly journal club that focuses on critical appraisal of methodology. Residents are also encouraged to take advantage of an optional research block during their third year of residency. Research throughout the year culminates in an annual Resident Research Day (RRD) where residents present and receive feedback on their work from other residents and faculty. Residents are expected to complete a project and present at four of five Resident Research Days during their five-year residency.

### Data collection

Data collected were divided into two five-year timeframes: pre (2002–2007) and post (2007–2012) residence research program implementation.

In addition to research activities known to the Division of General Surgery, we sought to identify additional works completed by the residents. A search was undertaken to identify abstracts published by residents at key national and international research meetings between 2002 and 2012. Key meetings were major conferences (both specialty and subspecialty) deemed by members of the division of general surgery to be meaningful, and included meetings of the Canadian Surgical Forum, Trauma Association of Canada, American Association for the Surgery of Trauma, American College of Surgeons, the Hepato-Pancreato-Billiary Association, and the American Society of Colorectal Surgery. Additionally, a search of major databases (PubMed, Embase, CINAHL) was conducted to identify all articles published over a 10-year period (2002–2012) by General Surgery Residents at the London Health Sciences Centre during their period of residency. Residents were searched individually and any works published during their period of residency, and in collaboration with other members of the division of general surgery, were recorded.

A database of all resident research presented at the annual Resident Research Day is updated each year. The database includes an indicator of the quality of presentations. Presentations that were approved by a faculty advisor, and that are neither a case study nor a research proposal, are considered of good quality. The number of research presentations meeting these criteria at the annual RRD was recorded by year.

### Resident survey

A survey of residents was conducted in 2007 (just prior to resident research program implementation), 2009, and 2015. This survey was developed by the resident research director, piloted with members of the residency training committee, and revised based on feedback. This survey asked questions pertaining to residents’ satisfaction with the current research program, and solicited feedback on their attitudes towards program goals and expectations. Residents were asked to indicate their level of agreement with a given statement. Surveys were sent to all residents electronically. Completed anonymous surveys were returned to the program administrator. Several reminders were sent to complete and return outstanding surveys following a modified Dillman approach.^20^ Survey responses were compiled by the program administrator into a database and provided to the resident research program director.

### Data summary and analysis

As much of these data were collected for ongoing program development and monitoring purposes, Research Ethics Board (REB) approval was not required for this study as determined upon consultation with the Western University REB (London, Ontario).

The total number of both published abstracts and publications by residents was calculated, as was the mean number of abstracts and publications per resident each year. Means with standard deviations (SD) were calculated to account for the change in number of residents each year, while providing an indication of overall productivity. The proportion of residents presenting at the annual RRD and the proportion meeting RRD criteria each year were also calculated.

To assess the overall change in productivity in pre vs. post resident research program periods, independent t-tests were performed to determine if there was a difference in the mean number of published abstracts and publications per resident for each five-year period (significance, p<0.05, 2 tailed). Data were also presented as a bar chart to provide a visual depiction of the change in the mean number of abstracts and publications each year. Similarly, Z-tests for independent proportions were performed to determine if a difference existed in the proportion of residents presenting at the annual RRD, as well as to assess any difference in the proportions of RRD presentations meeting criteria.

Results of the resident surveys were displayed graphically in bar charts. Answers were broken down into three categories: 1) general attitudes towards resident research, 2) motivation for conducting research, and 3) barriers to conducting resident research. Descriptive trends in responses over the three survey periods were compared.

## Results

Over the 10-year study period, resident research for 68 residents was evaluated. The total number of residents by year is indicated in Table 1.

**Figure 1:**
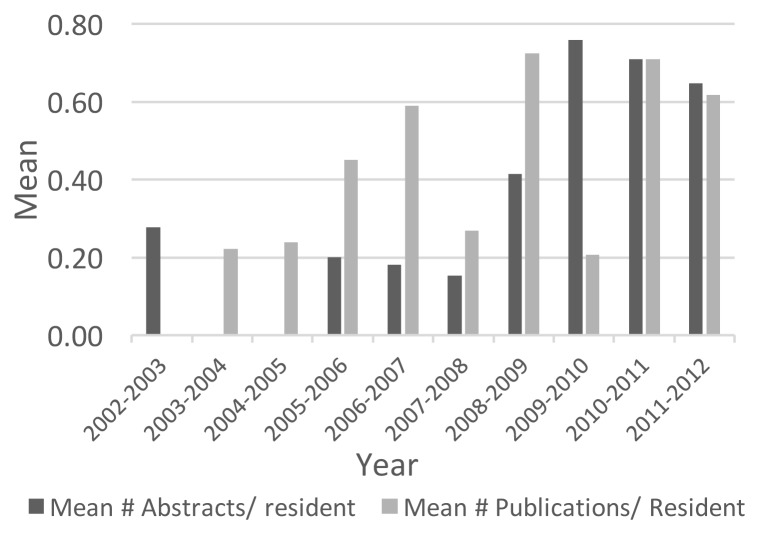
Mean number of published abstracts and publications per resident, by year

The mean number of published abstracts presented at national and international conferences each year increased significantly between pre and post resident research program time frames (0.13 (SD 0.13) vs. 0.54 (SD 0.25), respectively; p=0.013). The mean number of publications was greater post compared to pre resident research program implementation (0.51 (SD 0.25) vs. 0.30 (SD 0.23), respectively) but this was not statistically significant (p=0.21). The mean number of published abstracts and publications, by year, is presented in Figure 1.

Between pre and post resident research program time periods, the overall proportion of residents presenting at the annual RRD did not increase significantly (68% and 73%, respectively) over each five-year period. However, in each five-year period the proportion of presentations meeting RRD criteria did increase significantly following implementation of a program of resident research (70.6 vs. 98.2%, respective; p<0.001), suggesting that the quality of resident research presented at the annual RRD improved. A breakdown of RRD activities by year are presented in Table 1.

**Figure 2:**
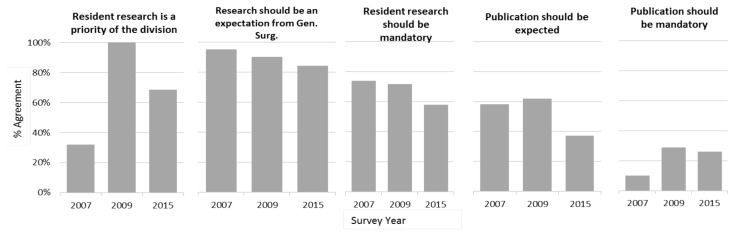
Survey responses of resident attitudes towards research

Attitudes towards resident research (Figure 2) have shifted somewhat over the three time periods. Although the consensus in 2015 was that research was a priority of the division, it was not felt as strongly that research should be mandatory or that publications should be expected. This is a large shift from 2007 in which there was very little perceived research support, but a stronger agreement with mandatory research and expected publication.

**Figure 3:**
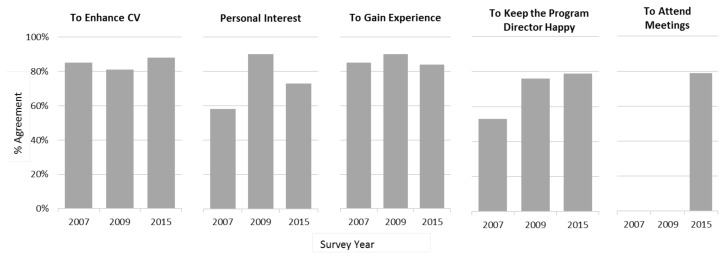
Survey responses of resident motivation for conducting research

Motivation for research (Figure 3) remained fairly constant across the three time points. Personal interest in research was higher in 2009 and 2015 than in 2007.

**Figure 4:**
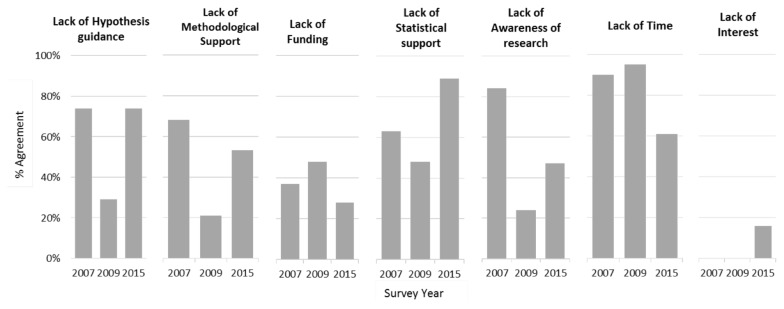
Survey responses of perceived barriers of conducting research

Perceived barriers to research (Figure 4) were also somewhat variable across years, however, there appears to be a general consensus that lack of time to conduct research is a barrier. This was particularly apparent in the 2007 and 2009 surveys.

## Discussion

The introduction of a formal resident research program in a Canadian General Surgery training program has resulted in an increase in research productivity. On average, the number of abstracts published increased significantly between pre and post resident research program time frames. The mean number of publications per resident also increased, although this was not statistically significant. Presentations at the local Resident Research Day improved, both in the proportion of residents presenting, as well as the quality of research presented. Resident attitudes towards research have changed somewhat over time.

In addition to an increase in research productivity, short term program goals were realized. Although the goal of 80% of residents presenting at the annual resident research day was not met, the proportion presenting did increase each year. Both personal and academic situations exempted some residents from presentation requirements (n=6). This would have reduced the total number of residents expected to present, thereby, increasing the proportion presenting at RRD. Furthermore, the quality of research presented improved dramatically with 100% of presentations meeting RRD criteria between 2008–2012, and 88% in 2007–2008. Although the abstract submission rate is unknown, there was an increase in the number of published abstracts over the study period. A statistically significant increase in published abstracts was also noted between pre and post program implementation. Finally, although we were not able to directly measure the number of papers submitted for peer reviewed publication, the mean number of publications per resident did increase following the implementation of the research program. Delays in the publication process may have affected the annual number of publications.

It is difficult to compare the success of this particular program of resident research to resident research programs at other institutions as structures and curricula, as well as program goals and expectations, vary widely. However, in keeping with the majority of programs, the research environment evaluated in the present study includes mentorship from a senior staff member,^2^,^4^–^7^,^15^ a classroom component with formal lectures and seminars on research related topics,^13^,^21^ and a resident research day to showcase resident led projects, and encourage the majority of individuals to present their work.^2^,^10^,^16^,^22^ Fewer programs include dedicated research time, as this particular program does, as an elective component.^3^,^12^,^21^ In almost all programs, including our program, presentation at local and national conferences and peer-reviewed publication were taken to be the ultimate measure of resident research success. Success may also be measured by the proportion of residents completing fellowships and choosing academic careers. We are considering using this additional indicator in future program evaluation studies.

It appears that although there was a change in attitudes towards research as different cohorts of residents moved through the program, this may not have had an effect on research productivity. Research activities in the form of published abstracts increase significantly before and after introduction of the research program, and the number of publications also increased. This is in keeping with much of the current research in which statistically significant improvements in the number of publications were often seen in these same areas. This may be a reflection of residents in later cohorts reporting that they felt well supported in research activities. However, in the 2015 survey, several residents indicated no interest in conducting any research. This general disinterest in research activities may be reflected in future reports of resident research activities. Throughout all three years of the resident survey, lack of time was consistently reported as a barrier to conducting research. This should be considered in the continued development of this and other resident research programs and strategies to ensure adequate and dedicated time is provided for conducting research activities.

This study is not without limitations. Firstly, we used a pre-post design. The longitudinal timeframe of this study meant that there were other changes to the General Surgery residency program itself which may have affected resident attitudes towards research. This included changes in the resident research program director. As observed in the resident survey, keeping the program director happy was a greater motivation to conducting research in 2009 and 2015 surveys (76 and 79%, respectively vs. 53% in 2007), reflecting a potential impact of the program director on resident attitudes towards research. Furthermore, changes in accreditation standards of residency programs may also have had an effect on resident expectations and research activities. In addition, the current program is compared to a poor control, i.e. no program at all. It can be argued that any educational intervention will likely impact outcomes, however, with no comparator intervention, the mechanism of the effect will be largely unknown.^23^ We cannot determine, therefore, whether a different educational program would have had larger benefits and/or fewer costs.^24^ This is also a concern with multifactorial interventions, such as the one described in this study, where determining which components of an educational program had the greatest impact can be difficult.^23^ Secondly, our outcome indicators suffer from inherent limitations. There can be a significant time lag between submission of manuscripts and publication. Resident works published outside of the study timeframe (but completed and submitted during the study timeframe) may have been missed. Conversely, resident publications may be related to work conducted before residency. Additionally, this may have implications with the denominator used to calculate the mean number of publications per resident each year. Furthermore, ongoing research is unaccounted. Residents involved in large research projects or studies with long timeframes may not have had the opportunity to present data or publish results in either abstract or manuscript form. Future program evaluation should examine the number of individual research projects being conducted each year to further assess overall increases in research activities. Finally, our evaluation focused on the intended outcomes. We did not examine potential unintended consequences on the residency program as a whole, such as resident satisfaction, burn-out, or performance in other competency domains.^24^

Results indicate that this program of resident research has been successful in increasing resident research activities. Although productivity increased, goals will be updated in order to move this program forward and further inspire research activities, specifically an increase in peer-reviewed publications. The curriculum document will be formalized and expanded in order to more clearly and fully delineate resident research expectations and updated program goals. Surveys of resident opinions will continue to provide feedback on attitudes towards research and this feedback will be incorporated into future goal setting and program development.

### Conclusion

The Resident Research program in the Division of General Surgery at the London Health Sciences Centres has been successful thus far. The setting of pre-defined, modifiable, and achievable goals, delineating clear resident research expectations, and the provision of tools to promote resident research success, have all contributed to the success of this program. This supports the notion that similar programs could be effective at other institutions and departments across the Canadian medical resident education system. Ongoing evaluation of this and similar programs should be conducted to ensure continued success of such initiatives.

## Figures and Tables

**Table 1:** Summary of Resident Research Day activities

Years	# Residents/year	Residents presenting at Resident Research Day % (n)	Presentations meeting RRD criteria % (n)
**Pre Resident Research Program**			
2002–2003	18	83 (15)	60 (9)
2003–2004	18	67 (12)	58 (7)
2004–2005	21	67 (14)	86 (12)
2005–2006	20	65 (13)	77 (10)
2006–2007	22	64 (14)	71 (10)
**Post Resident Research Program**			
2007–2008	26	62 (16)	88 (14)
2008–2009	29	76 (22)	100 (22)
2009–2010	29	72 (21)	100 (21)
2010–2011	31	77 (24)	100 (24)
2011–2012	34	79 (27)	100 (27)

***Note:*** Resident Research Day, RRD
